# Burosumab treatment in a child with cutaneous skeletal hypophosphatemia syndrome: A case report

**DOI:** 10.1016/j.bonr.2021.101138

**Published:** 2021-10-01

**Authors:** Manal Khadora, M. Zulf Mughal

**Affiliations:** a Department of Pediatric Endocrinology, Latifa Hospital, Oud Metha Road, Al Jaddaf, Dubai, United Arab Emirates; b Department of Pediatric Endocrinology, Royal Manchester Children's Hospital, Manchester University NHS Foundation Trust, Oxford Road, Manchester M13 9WL, UK; c Faculty of Biology, Medicine & Health, University of Manchester, Manchester M13 9PL, UK

**Keywords:** Cutaneous skeletal hypophosphatemia syndrome, Hypophosphatemic rickets, Fibroblast growth factor 23, Burosumab

## Abstract

Cutaneous skeletal hypophosphatemia syndrome (CSHS) is a rare disorder caused by somatic mosaicism for the gain of function *RAS* mutations . Affected patients have segmental epidermal nevi, dysplastic cortical bony lesions, and fibroblast growth factor-23 (FGF23)–mediated hypophosphatemic rickets. Herein, we describe a case of an Emirati girl with CSHS, whose hypophosphatemic rickets and osteomalcic pseudofractures and dysplastic bony lesions failed to recover due to poor adherence to treatment with oral phosphate supplements and alfacalcidol (conventional treatment). Treatment with burosumab, a fully human immunoglobulin G1 monoclonal antibody against FGF23 for 12 months, led to normalization of serum inorganic phosphate and alkaline phosphatase levels, radiographic healing of rickets, partial healing of pseudofractures, improvement in 6-minute walk test, and the physical scale of the Pediatric Quality of Life Inventory. We conclude that burosumab is effective in treatment of CSHS, however results of the ongoing phase 2 trial in adults (NCT02304367) are awaited.

## Highlights

• Cutaneous skeletal hypophosphatemia syndrome (CSHS) is caused by somatic gain-of-function RAS mutations.
• It is associated with fibroblast growth factor-23 (FGF23) mediated hypophosphatemic rickets.
• Burosumab, a monoclonal antibody to FGF23 may have a role in the treatment of CSHS.

## 1 Introduction

Epidermal nevus syndrome (ENS) is a heterogeneous rare skin disorder characterized by segmental epidermal nevi. The condition is caused by somatic mosaicism for the gain of function mutations in members of the *RAS* subfamily, and is often associated with non-cutaneous abnormalities that frequently affect the brain, eye, and skeletal systems. When ENS is associated with hypophosphatemic rickets or osteomalacia, it is known as cutaneous skeletal hypophosphatemia syndrome (CSHS) (Lim et al., 2014; Ovejero et al., 2016; Lim et al., 2016; de Castro et al., 2020). Patients with CSHS have focal dysplastic cortical bone lesions containing cells with somatically active *RAS* mutations that lead to excessive plasma fibroblast growth factor 23 (FGF23) levels (Lim et al., 2014; Ovejero et al., 2016; Lim et al., 2016; de Castro et al., 2020). Plasma FGF23 is an essential regulator of phosphate and vitamin D metabolism. However, elevated plasma levels of FGF23 downregulate the expression of sodium-dependent phosphate reabsorption cotransporters (Npt-2a and Npt-2c) in the proximal renal tubules, leading to urinary phosphate wastage (Beck-Nielsen et al., 2019). FGF23 also reduces renal 1α-hydroxylase activity and stimulates expression of 24-hydroxylase leading to lower serum 1,25-dihydroxy vitamin D (1,25(OH)2D) levels that impair the gastrointestinal absorption of inorganic phosphate (iP) and calcium (Beck-Nielsen et al., 2019). The net effect is reduced serum iP levels that are important in the pathogenesis of rickets and osteomalacia (Mughal, 2002). Additionally, skeletal manifestations of CSHS include fractures, limb deformities, and scoliosis (Ovejero et al., 2016).

Burosumab, a fully human immunoglobulin G1 monoclonal antibody against FGF23, has been approved for the treatment of children with X-linked hypophosphatemia (XLH), a multisystem disorder caused by increased expression of FGF23 (Burosumab summary of product characteristics (Burosumab summary of product characteristics (Burosumab SmPC), n.d., Al Juraibah et al., 2021). Burosumab binds to and inhibits the excessive biological activity of FGF23, which helps restore the renal tubular reabsorption of phosphate and intestinal absorption of calcium and phosphate (by increasing serum 1,25(OH)2D levels), and thus, improves skeletal mineralization. In clinical trials with children aged 1–12 years with XLH, burosumab increased the serum levels of inorganic phosphate (iP) and alkaline phosphatase (ALP), healed rickets, increased linear growth, and improved mobility, despite prior treatment with phosphate salts and activated forms of vitamin D (calcitriol/alfacalcidol) (Carpenter et al., 2018; Whyte et al., 2019; Imel et al., 2019).

The USA Food and Drug Administration have approved Burosumab for the treatment of XLH in children and adults from the age of 6 months onwards, and by the European Medicines Authority (Burosumab SmPC) in children from the age of 1 year and adolescents with growing skeletons.

Herein, we have described a case of an Emirati girl with CSHS whose FGF23-mediated hypophosphatemic rickets, osteomalcic pseudofractures, and dysplastic cortical skeletal lesions failed to heal due to poor adherence to treatment with oral phosphate supplements and alfacalcidol (1α-hydroxycholecalciferol). The patient was treated with burosumab for 12 months, which led to normalization of serum iP and ALP levels, radiographic healing of rickets, partial healing of the pseudofractures, improvement in 6-minute-walk test, and physical scale of the Pediatric Quality of Life Inventory.

## 2 Case presentation

**Fig. 1:**
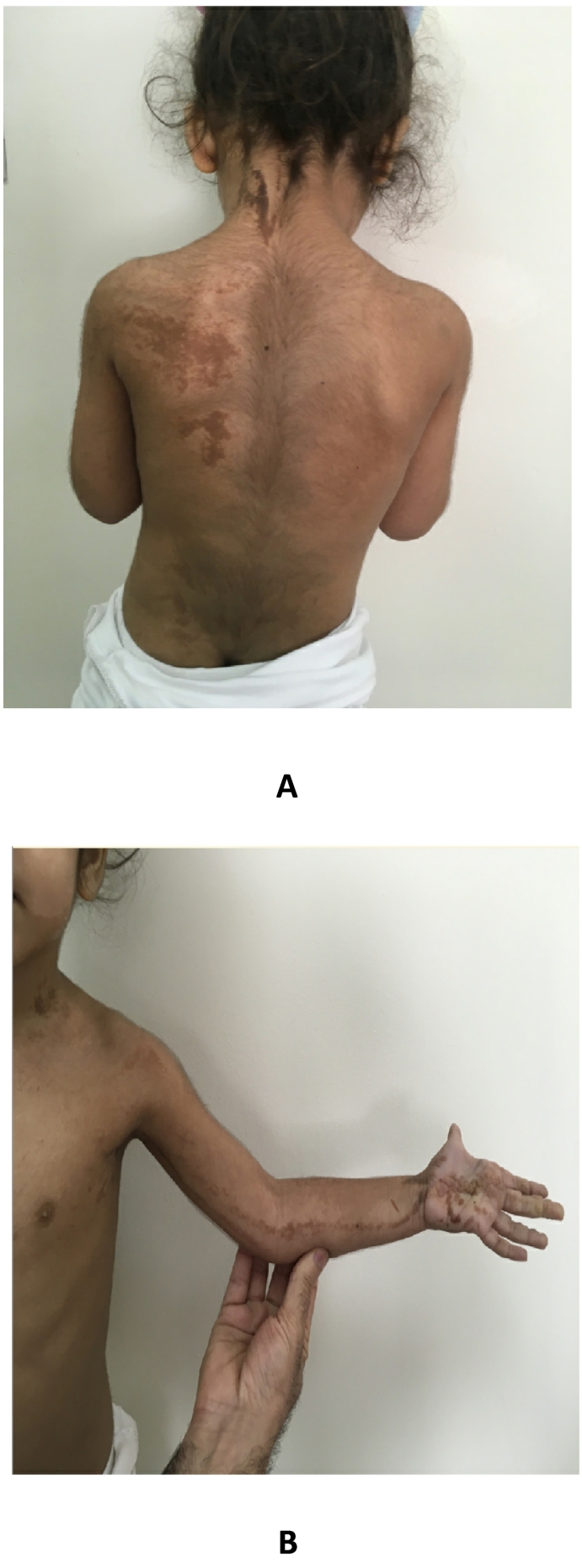
Photographs of the patient showing linear epidermal nevi distributed on the left side of her body.

The patient is the fifth child born to second-degree consanguineous Emirati parents. She was born by emergency cesarean section due to fetal distress at 36-week gestation and weighed 2.6 kg (−1.5 SDS) at birth. She has four healthy siblings, but one of them underwent treatment for vitamin D deficiency rickets during early childhood. The patient had an extensive unilateral hyperpigmented epidermal nevus on the left side of the body involving the scalp, forehead, mandible, middle of tongue, neck, shoulder, upper limb, trunk, and buttock area (Fig. 1A–B). Investigations of the heart murmur revealed that she had an atrial septal defect. Additionally, the child's mother noticed leg length discrepancy when she was 1-year-old.

She was referred to the Pediatric Endocrine unit at the Latifa Women and Children Hospital in March 2018, at the age of 3 years and 10 months, to manage rickets and leg length discrepancy. She had been treated with oral phosphate supplement (Phosphate Sandoz) and alfacalcidol at the peripheral hospital for 10 months. The parents reported that she had reduced energy levels and impaired mobility, and often required a stroller. However, her development was normal, and unlike some patients with CSHS, she did not suffer from seizures.

The initial clinical examination at her first visit to the Pediatric Endocrinology clinic in March 2018 revealed 13 kg weight (7th centile; −1.48 SDS) and 100.5 cm height (58th centile; 0.20 SDS). In addition to the giant unilateral hyperpigmented epidermal nevus, she had extensive dental enamel hypoplasia, and her left leg was thinner and approximately 1 cm shorter than the right leg. Further, the initial investigations after discontinuing the treatment (washout period) for 3 days revealed low serum iP and high serum ALP levels than normal for her age (Table 1). The adjusted serum levels of calcium (Ca), parathyroid hormone (PTH), 25-hydroxyvitamin D (25OHD), and 1,25 (OH)2D are shown in Table 1. The patient's maximal reabsorption rate of phosphate per unit volume of glomerular filtrate (TmP/GFR) was lower than normal for her age. The plasma c-terminal FGF23 levels were inappropriately elevated along with increased urinary phosphate excretion than normal (Table 1).

**Table 1:** Biochemical parameters in March 2018, after the first washout period (3 days). Reference values are shown in parentheses.

Biochemical parameters	Values
Serum calcium mg/dl (8.4–10.6)	9.5
Fasting serum phosphate mg/dl (3.4–5.5)	2.9
Alkaline phosphatase U/L (<281)	548
Urine TmP/GFR mg/dl (4–8)	2.8
Intact PTH pg/ml (15–65)	12.5
25-Hydroxy vitamin D ng/ml (20–60)	35.3
1,25-Dihydroxyvitamin D pg/ml (30–90)	30.4
C-terminal fibroblast growth factor-23 RU/ml (39–91)	479

TmP/GFR, maximal reabsorption rate of phosphate per unit volume of glomerular filtrate; PTH, parathyroid hormone.

**Fig. 2:**
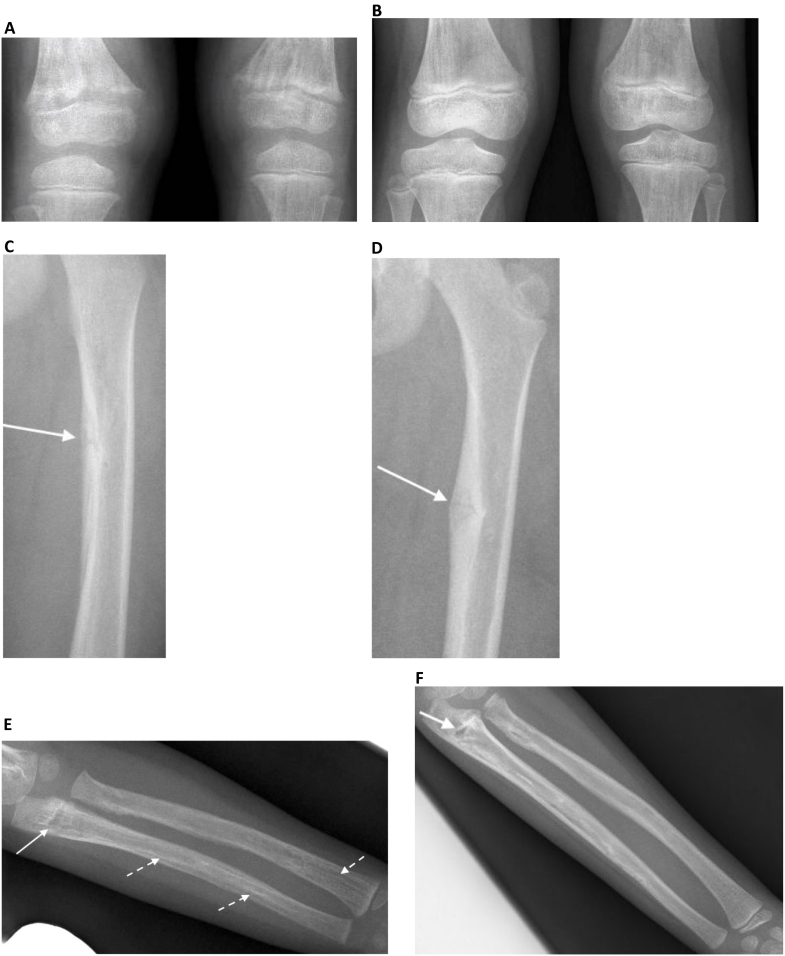
Radiographic comparison of patient's condition after 10 months of conventional therapy and before treatment with burosumab. A. Radiographs of the knees before treatment with burosumab. Widening and fraying of the distal femoral metaphyses are consistent with rickets. Moreover, the sub-metaphyseal areas of lucency and vertical cortical striations are clearly visible in the left distal femoral radiograph. B. Radiographs of knees 12-months after treatment with burosumab. Radiograph of knees showing healing of radiological signs of rickets and improvement in bone texture. C. Radiograph of the left proximal femur before treatment with burosumab. Note the left medial cortical pseudofracture (arrow), with periosteal reaction. D. Radiograph of the left proximal femur 12 months after treatment with burosumab. Note cortical thickening and partial healing of left medial cortical pseudofracture (arrow). E. Radiograph of the left forearm before treatment with burosumab. Note the proximal ulnar pseudofracture (arrow). Also shown are diaphyseal areas lucency and sclerosis (dashed arrows). F. Radiograph of the left forearm 12-months after treatment with burosumab. Note the partial healing of the proximal ulnar pseudofracture (arrow) and improvement in the diaphyseal bone texture.

The skeletal investigation showed radiological changes confirming rickets: widening and fraying of the metaphyses that were clearly visible at the distal end of the femurs (Fig. 2A). Additionally, there were pseudofractures in the left proximal left femur and left proximal ulna (Fig. 2C, E). There was an abnormal bone texture, with areas of lucency and sclerosis within diaphysis (Fig. 2C, E).

The patient was diagnosed with CSHS with FGF23-mediated hypophosphatemic rickets due to increased urinary phosphate excretion and hypophosphatemia along with normal serum PTH levels and inappropriately elevated plasma c-terminal FGF23 levels. A whole-exome sequencing analysis of cells from the nevoid skin biopsy revealed a somatic missense variant c.182A>G p.(Gin61Arg) (chr11:533874;hg19) in the *HRAS* (OMIM *190020; chromosome 11p15.5). However, this mutation was not identified in the DNA extracted from the patient's circulating leucocytes.

Therefore, she was treated with phosphate Sandoz 500 mg twice daily, alfacalcidol 500 ng (5 drops daily), and Cholecalciferol 600 IU daily. However, due to gastrointestinal side effects of the phosphate salts, her adherence to treatment with conventional therapy for hypophosphatemic rickets was poor. During the 12 months of conventional treatment, the patient's serum ALP levels remained elevated than normal for her age, and follow-up radiographs showed incomplete healing of rickets and pseudofractures; also, her lethargy persisted.

Further, we obtained approval from the hospital's Medicine Management Board, and treated the patient with burosumab at an initial dosage of 0.8 mg/kg body weight, injected subcutaneously every two weeks, after discontinuing the conventional therapy (washout period) for one week. The patient's treatment and monitoring protocol was based on the burosumab (Crysvita) summary of product characteristics (SmPC; https://www.ema.europa.eu/en/documents/product-information/crysvita-epar-product-information_en.pdf).

A 6-minute-walk test was conducted under the supervision of a physiotherapist before starting the treatment with burosumab and at six and 12 months of treatment. The UK-English version of the Pediatric Quality of Life Inventory™ 4.0 (PedsQL4) was used to measure the health-related quality of life before and after 12-months of therapy. The patient's biochemical parameters, and the outcome of 6-minute walk test and PedsQL4, following the second washout period (7 days) and after burosumab treatment are shown in Table 2. After the first two doses, the burosumab dose was titrated down to 0.4 mg/kg because of the high serum iP level of 6.1 mg/dl (normal range, 3.4–5.5 mg/dl). The treatment protocol with dose titration to maintain the fasting serum iP level near the lower end of the age-related reference interval was continued for 12 months. She required burosumab dose of 5 mg (0.3 mg/kg to 0.4 mg/kg) throughout the treatment duration of 12 months, from the fourth-week onwards. She continued to receive burosumab subcutaneously every two-weeks at the hospital to ensure treatment compliance.

**Table 2:** Summary of biochemical parameters (reference values are shown in parentheses), and outcome of the 6-minute walk test and PedsQL4.

Parameters	Before start of burosumab, after 3-day washout period (March 2018)	Twelve months after treatment with burosumab (August 2020)
Serum calcium in mg/dl (8.4–10.6)	9.5	9.6
Fasting serum phosphate in mg/dl (3.4–5.5)	2.6	5.0
Alkaline phosphatase in U/L (<281)	638	300
Urine TmP/GFR mg/dl (4–8)	2.1	4.41
Intact parathyroid hormone in pg/ml (15–65)	17.9	20.5
25-Hydroxy vitamin D in ng/ml (20–60)	31.4	35.6
1,25-Dihydroxyvitamin D in pg/ml (30–90)	33.7	39.5
C-Terminal fibroblast growth factor-23 RU/ml (39–91)	479	–
6-min walk test in meters^a^	292 m (−2.3 SD)	388.62 (−1.25 SD).
PedsQL4 questionnaire (UK) version 4.0^b^		
Physical scale	43.75 (−2.56 SD)	81.25 (−0.23 SD)
Emotional scale	100 (+1.43 SD)	100 (+1.43 SD)
Social scale	80 (−0.26 SD)	80 (−0.26 SD)
School scale	100 (+1.20 SD)	100 (+1.20 SD)

PedsQL4, Pediatric Quality of Life Inventory™ 4.0; TmP/GFR, maximal reabsorption rate of phosphate per unit volume of glomerular filtrate.

a SD calculated from 6 min walk test normal values provided in (Geiger et al., 2007).

b SD calculated from PedsQL4 questionnaire (UK) version normal values provided in (Upton et al., 2005).

**Fig. 3:**
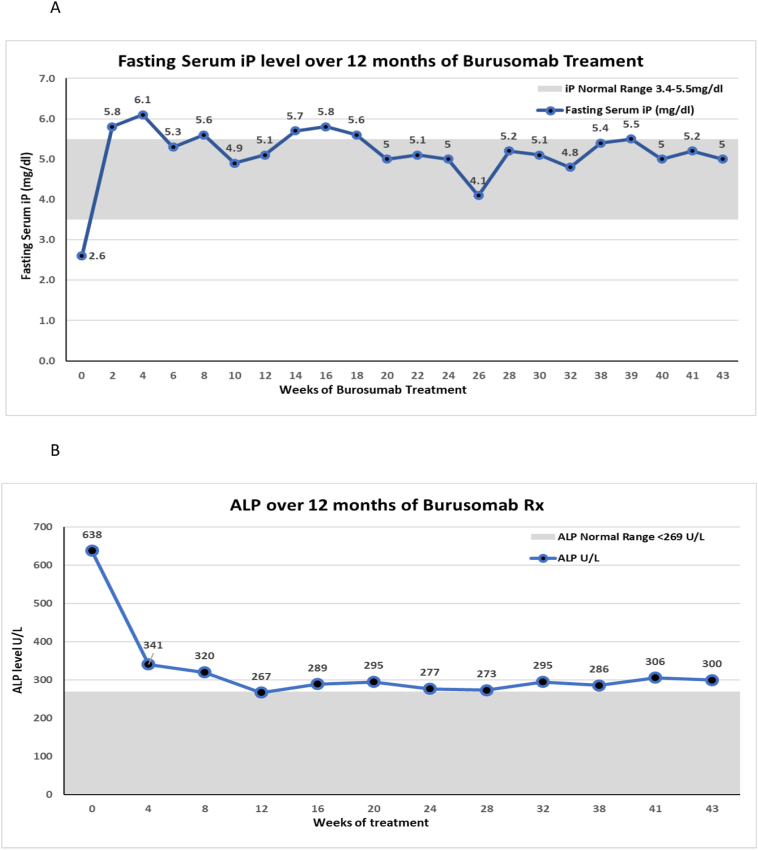
Fasting serum levels of phosphate (iP) and alkaline phosphatase (ALP). A. Fasting serum iP levels for 12-months of treatment with burosumab. B. Serum ALP levels for 12-months of treatment with burosumab.

As shown in Fig. 3A–B, the patient's serum iP and ALP levels normalized after the first three doses of burosumab and continued to remain normal throughout the treatment period. She grew normally with her body height and weight SDS along the 50th and 25th centile, respectively.

As shown in Table 2, patient's biochemical parameters, the 6-minute-walk test, and physical scale sore for the PedsQL4 questionnaire showed significant improvement after completion of one-year of treatment with burosumab. Follow-up radiographs of the lower and upper limbs showed radiological healing of rickets (Fig. 2B), partial healing of pseudofractures, and improvement in the texture of diaphysis (Fig. 2D, F). The patient did not develop an injection site or general adverse events during the period of treatment with burosumab.

## 3 Case discussion

We have described the case of a girl with CSHS, whose hypophosphatemic rickets, pseudofractures, and dysplastic bony lesions (abnormal diaphyseal areas of lucency and sclerosis) failed to heal with “conventional treatment,” comprising phosphate salts and alfacalcidol, due to poor adherence to the treatment. Twelve months of treatment with burosumab, administered subcutaneously every two weeks, led to the normalization of the biochemical parameters (especially iP, TmP/GFR, and ALP), healing of rickets, partial healing of pseudofractures, and improvement in the dysplastic skeletal lesions. The treatment also improved the outcome of the 6-minute walk test and patient-related health outcomes (assessed by PedsQL4). To the best of our knowledge, this is the first study to report successful treatment of CSHS with burosumab.

CSHS is a rare disorder caused by de-novo somatic mosaicism for the gain of function mutations in the *RAS*, frequently in *HRAS* or *NRAS* (Ovejero et al., 2016). Moreover, the whole-exome sequencing of DNA extracted from the patient's nevoid skin biopsy revealed a somatic missense variant in the *HRAS*. In addition to pigmented skin lesions, and abnormalities in the brain and eyes, CSHS is often associated with dysplastic cortical skeletal lesions and FGF23-mediated hypophosphatemic rickets and osteomalacia. These bony lesions are usually present ipsilateral to the nevoid skin lesions (Heike et al., 2005), similar to that in our case. Other skeletal manifestations of CSHS include fractures, limb deformities, and scoliosis. Limited bone biopsy studies of the focal dysplastic cortical bone lesions in patients with CSHS have shown the replacement of the normal lamellar bone with focal regions containing irregular fibroblast-like spindle-shaped cells in a relatively dense collagen matrix (Lim et al., 2016). These focal changes also have extensive unmineralized osteoid, characteristic of osteomalacia (de Castro et al., 2020). The DNA from the dysplastic bone lesions, but not healthy lamellar bone tissue, harbor the same *HRAS* or *NRAS* mutations that are found in DNA extracted from the nevoid skin cells or circulating leucocytes (Lim et al., 2016). It is known that gain-of-function mutations in members of the *RAS* signaling pathway cause the increased expression of FGF23 through increased activation of the fibroblast growth factor receptor 1 (de Castro et al., 2020). There are reports of partial improvement in rickets and osteomalacia in patients with ENS following debulking of the nevoid skin lesions (Aschinberg et al., 1977; Ivker et al., 1997; Saraswat et al., 2003; Sanmaneechai et al., 2006).However, the complete removal of nevoid lesions is often not feasible in cases with extensive skin lesions. Some patients were also treated with phosphate salts and activated forms of vitamin D in a previous study (Ramesh et al., 2017). Recent studies have shown that the nevoid skin lesions are not the source of production of FGF23 in patients with CSHS. Therefore, it is possible that activating mutations in *RAS* in the dysplastic skeletal lesions are the cause of elevated FGF23 levels in patients with CSHS (Ovejero et al., 2016; Narazaki et al., 2012). The elevated levels of plasma FGF23 leads to development of rickets and osteomalacia.

Conventional therapy with phosphate salts and calcitriol/alfacalcidol leads to the healing of hypophosphatemic rickets and osteomalacia in patients with CSHS (Ovejero et al., 2016; Sukkhojaiwaratkul et al., 2014; Moreira et al., 2010; Welfringer-Morin et al., 2020). However, adherence to conventional therapy is often limited by the requirement of daily administration of phosphate salts and occurrence of gastrointestinal symptoms. In children with XLH, this treatment regimen is also associated with the risk of developing secondary hyperparathyroidism and nephrocalcinosis. Finally, conventional therapy fails to correct the underlying pathogenesis of XLH, normalize fasting serum iP levels, and fully heal rickets (Imel et al., 2019). Thus, burosumab, a fully human immunoglobulin G1 monoclonal antibody against FGF23, serves as the targeted therapy in XLH and other hypophosphatemic disorders arising from excess serum FGF23 levels. In a recent phase 3 clinical trial in children with XLH, treatment with burosumab, at a dose of 0.8 mg/kg administered subcutaneously every 2 weeks, normalized phosphate homeostasis (iP and TmP/GFR), serum ALP levels, and improved rickets, growth, bowing, and mobility, than in those who continued to receive conventional therapy (Imel et al., 2019). Burosumab is currently only licensed for the treatment of XLH and tumor-induced osteomalacia (TIO). Jan de Beur et al. presented data on phase 2 clinical trial for burosumab treatment in 14 adult patients with TIO and one patient with CSHS at the 2017 American Society for Bone and Mineral Research annual meeting (Jan de Beur et al., 2021). Treatment with burosumab for 24 weeks improved the serum levels of iP and 1,25(OH)2D, TmP/GFR, and lower limb strength. Burosumab also decreased pain and fatigue in the patients (Jan de Beur et al., 2021).

The bespoke treatment and monitoring protocol based on the burosumab (Crysvita) summary of product characteristics (Burosumab SmPC) was approved by the Latifa hospital's Medicine Management Board. Burosumab treatment in our patient was well-tolerated without an injection site or other adverse effects reported during the 12-month treatment period. In addition to normalizing the patient's biochemical parameters of phosphate metabolism, treatment with burosumab normalized serum ALP levels and led to healing of rickets. The treatment also resulted in partial recovery of pseudofractures, possibly via healing of osteomalacia. Moreover, treatment with burosumab resulted in improved dysplastic bone lesions in the patient. Finally, whether the healing of dysplastic bone lesions that are the possible sources of FGF23 synthesis (de Castro et al., 2020), can modify the course of the bone disease in the patient, will become apparent if the requirement for burosumab dose in mg/kg decreases during the continuing period of monitoring of the patient.

Our treatment protocol was based on the Burosumab SmPC for the treatment of XLH, which recommends starting dose in children and adolescents of 0.8 mg/kg body weight, administered every two weeks. We had to de-escalate the dose to 0.3 to 0.4 mg/kg every 2 weekly. We speculate that this may be due to reduced production of FGF23 from focal dysplastic cortical bone lesions in CSHC, in comparison to children with XLH. Based on this experience, we would recommend that burosumab in children with CSHS should be started at the dose of 0.4 mg/kg, subcutaneously, every two weeks. The dose titration should be performed with the aim of maintaining fasting serum P levels around the lower end of the reference range for the age of the child.

## 4 Conclusion

We have described the case of a girl with CSHS whose hypophosphatemic rickets, osteomalcic pseudofractures, and dysplastic skeletal lesions failed to heal due to poor adherence to treatment with conventional therapy. Treatment of the patient with burosumab for 12 months normalized the serum iP and ALP levels, healed rickets, partially healed the pseudofractures, and improved the symptoms of myopathy and quality of life. We conclude that burosumab is effective in treatment of CSHS; results of the phase 2, open label, dose-finding study of burosumab trial in adults with CSHS (NCT02304367) are awaited.

## Consent

The parents of the patient provided written consent.

## Funding

This research did not receive funding from public, commercial, or not-for-profit sectors. MM and MZM designed the study protocol and wrote the manuscript.

## CRediT authorship contribution statement

MZM has received fees from Kyowa Kirin International, manufacturer of burosumab, for advisory boards and lectures. MM declares that she has no competing interest.

## Declaration of competing interest

Both the authors were equally involved in writing & editing the manuscript.
